# The Phosphatase PP2A Interacts With ArnA and ArnB to Regulate the Oligomeric State and the Stability of the ArnA/B Complex

**DOI:** 10.3389/fmicb.2020.01849

**Published:** 2020-08-21

**Authors:** Xing Ye, Marian Samuel Vogt, Chris van der Does, Wolfgang Bildl, Uwe Schulte, Lars-Oliver Essen, Sonja-Verena Albers

**Affiliations:** ^1^Molecular Biology of Archaea, Institute of Biology II, University of Freiburg, Freiburg, Germany; ^2^Department of Chemistry, Philipps University Marburg, Marburg, Germany; ^3^Institute of Physiology, Faculty of Medicine, University of Freiburg, Freiburg, Germany; ^4^Center for Biological Signaling Studies(BIOSS), Freiburg, Germany; ^5^Center for Integrative Signaling Studies (CIBSS), Freiburg, Germany; ^6^Loewe Center for Synthetic Microbiology, Marburg, Germany

**Keywords:** Crenarchaea, archaellum, archaellum regulation, protein phosphorylation, protein phosphatases, protein interaction

## Abstract

In the crenarchaeon *Sulfolobus acidocaldarius*, the archaellum, a type-IV pilus like motility structure, is synthesized in response to nutrient starvation. Synthesis of components of the archaellum is controlled by the archaellum regulatory network (arn). Protein phosphorylation plays an important role in this regulatory network since the deletion of several genes encoding protein kinases and the phosphatase PP2A affected cell motility. Several proteins in the archaellum regulatory network can be phosphorylated, however, details of how phosphorylation levels of different components affect archaellum synthesis are still unknown. To identify proteins interacting with the *S. acidocaldarius* phosphatases PTP and PP2A, co-immunoprecipitation assays coupled to mass spectrometry analysis were performed. Thirty minutes after growth in nutrient starvation medium, especially a conserved putative ATP/GTP binding protein (Saci_1281), a universal stress protein (Saci_0887) and the archaellum regulators ArnA and ArnB were identified as highly abundant interaction proteins of PP2A. The interaction between ArnA, ArnB, and PP2A was further studied. Previous studies showed that the Forkhead-associated domain containing ArnA interacts with von Willebrand type A domain containing ArnB, and that both proteins could be phosphorylated by the kinase ArnC *in vitro.* The ArnA/B heterodimer was reconstituted from the purified proteins. In complex with ArnA, phosphorylation of ArnB by the ArnC kinase was strongly stimulated and resulted in formation of (ArnA/B)_2_ and higher oligomeric complexes, while association and dephosphorylation by PP2A resulted in dissociation of these ArnA/B complexes.

## Introduction

Protein phosphorylation is one of the most important post-translational modifications in the three domains of life (Mok et al., 2010; Pereira et al., 2011; Reimann et al., 2013). The reversible character allows modulating protein properties in different cellular processes, including growth, division, metabolism, motility and immunity (Wurzenberger and Gerlich, 2011; Hempel et al., 2012; Arthur and Ley, 2013), which can help cells to rapidly transduce external and internal signals in a constantly changing environment. Bacteria, Archaea, and Eukarya can modify their proteins by phosphorylating them at serine, threonine, and tyrosine residues. Phosphorylation of histidines and aspartates is also found in Bacteria, Euryarchaeota and some Eukarya, where it functions in two-component signal transduction systems (TCS), but is lacking in Crenarchaeota and Nanoarchaeota (Esser et al., 2016). In addition, protein phosphorylation is also found for cysteine, lysine and arginine residues (Matthews, 1995; Khoury et al., 2011; Mijakovic et al., 2016).

Large-scale phosphoproteomic analysis is a widely used approach to understand the role of protein phosphorylation in different cellular processes. In the Euryarchaeon *Halobacterium salinarum*, a genome-wide phosphoproteomic analysis revealed 69 Ser/Thr/Tyr-phosphoproteins, which was the direct proof of Ser/Thr/Tyr phosphorylation existing in Archaea (Aivaliotis et al., 2009). Later, in the Crenarchaeon *S. solfataricus* P2, 540 unique Ser/Thr/Tyr-phosphoproteins were likewise identified by phosphoproteomic analysis, suggesting an important regulatory role of protein phosphorylation in *S. solfataricus* (Esser et al., 2012). Cultivation of *S. solfataricus* cells with different carbon sources (glucose and tryptone) caused a significant change of phospho-protein patterns, especially proteins involved in central carbohydrate metabolism, which suggested a role of protein phosphorylation in regulating the carbon flux. In *S. acidocaldarius*, phosphoproteomic studies revealed 801 unique phosphoproteins involved in most cellular processes (Reimann et al., 2013). Notably, the ratio of pTyr to pSer/pThr post-translational modification was much higher in *S. solfataricus* and *S. acidocaldarius* than in Bacteria and Eukarya, which suggested tyrosine phosphorylation plays a much more important role in *Sulfolobales*, possibly to complement the absence of TCS.

Protein phosphorylation in *S. acidocaldarius* is relatively well studied with a focus on its role in the archaellum regulatory network. The archaellum of *S. acidocaldarius* is encoded by the *fla* operon containing seven genes, which is under control of two promoters: the main inducible promoter *flaB* and a second weak constitutive promoter *flaX* (Lassak et al., 2013). Once cells sense a nutrient starvation signal, the *flaB* promoter is activated, followed by the transcription of the archaellum genes. Up to now, several regulators involved in archaellum regulation have been identified. The paralogs ArnR and ArnR1 are membrane-bound positive regulators whose genes are located upstream and downstream of the *fla* operon, respectively (Lassak et al., 2013). Deletion of *arnR* and *arnR1* reduced *flaB* expression and impaired swimming motility. Previous experiments showed that ArnR and ArnR1 regulated the *flaB* promoter, but not the *flaX* promoter (Lassak et al., 2013; Bischof et al., 2019). AbfR1, an archaeal biofilm regulator, is also a positive regulator for archaellum expression and its binding to DNA is phosphorylation dependent (Orell et al., 2013; Li et al., 2017). The forkhead associated (FHA) domain-containing protein ArnA and the von Willebrand factor type A (vWA) domain-containing protein ArnB, are two negative regulators for archaellum expression, and their deletion resulted in a significant increase of archaellum gene expression (Reimann et al., 2012). These two proteins strongly interacted with each other both *in vitro* and *in vivo*. The expression of *arnA* and *arnB* is kept constant in cells, while their interaction sequentially diminished in nutrient starved cells (Hoffmann et al., 2019). In *S. tokodaii*, ST0829, the homolog of ArnA, could bind to the *flaX* promoter region specifically (Duan and He, 2011), which suggested that ArnA might be involved in the archaellum expression by binding to the promoters of the *fla* operon. In *S. tokodaii*, ePK (Hanks-type protein kinases) ST1565 physically interacted with a FHA-domain-containing protein, ST0829, and phosphorylation by ST1565 could negatively regulate ST0829’s interaction with the promoter region of archaellum protein-encoding operon (Wang et al., 2010; Duan and He, 2011).

Eleven ePKs and aPKs (non-canonical Hanks-type protein kinases) are encoded in the *S. acidocaldarius* genome. Deletion of *saci_0965*, *saci_1181* (*arnS*), and *saci_1193* (*arnC*) reduced motility, while deletion of *saci_1694* (*arnD*) caused hypermotility of cells (Haurat et al., 2017; Hoffmann et al., 2017). *In vitro*, ArnC phosphorylates ArnA and ArnB, while ArnD specifically phosphorylates ArnB (Reimann et al., 2012; Hoffmann et al., 2019). PTP (tyrosine phosphatase) and PP2A (serine/threonine phosphatase) are the only two protein phosphatases in *S. acidocaldarius* (Reimann et al., 2013). Deletion of *saci_pp2a* resulted in a significant increase of motility and changes in growth, cell shape, and cell size. No such effects were observed in the PTP deletion mutant. In addition, a total of 155 differentially expressed genes were identified in Δ*saci_ptp* and Δ*saci_pp2a* by transcriptomic analysis, including genes encoding components of the archaellum, components of the respiratory chain and transcriptional regulators. Phosphoproteomic analysis obtained 551 specific phosphoproteins in Δ*saci_ptp* including AbfR1, ArnB, ArnR1, and FlaJ, and 387 specific phosphoproteins in Δ*saci_pp2a* specific phosphoproteins including FlaJ. The phosphorylation state of Tyr 84 and Ser 87 of AbfR1 regulates the interaction with its own promoter and the regulatory function in biofilm formation and motility (Li et al., 2017). In addition, a recent study indicated that the archaellum regulator ArnR and ArnR1 are phosphorylated by ArnC *in vitro*, however, whether the phosphorylation was involved in regulating their functions was not demonstrated yet (Bischof et al., 2019).

In this study, we set-out to unravel the roles of PTP and PP2A in the archaellum regulatory network by identifying the proteins which interact with PTP and PP2A. ArnA and ArnB were identified as important proteins interacting with PP2A, and their interaction and their dependence on the phosphorylation state of the different components were analyzed *in vitro*. This work would help us better understand how protein phosphorylation is involved in the regulation of expression of the archaellum in *S. acidocaldarius*.

## Materials and Methods

### Strains and Growth Conditions

*Sulfolobus acidocaldarius* MW001 and derived mutants used in this study (Supplementary Table S1) were cultivated at 75°C and 120 rpm in Brock’ basal medium (pH3.0–3.5) supplemented with 0.1% (w/v) NZ-amine, 0.2% (w/v) dextrin and 10 μg/mL uracil (Brock et al., 1972; Wagner et al., 2012). *Escherichia coli* strains used in this study (Supplementary Table S1) were grown in Lysogeny broth (LB) or on LB agar plates at 37°C and ampicillin (50 μg/mL), kanamycin (25 μg/mL) or chloramphenicol (30 μg/mL) were added when needed. *E. coli* Top 10 was used for plasmid propagation. *E. coli* ER1821 was used to methylate plasmids for transformation into *S. acidocaldarius*. *E. coli* Rosetta (DE3)/pLysS was used for overexpression of *S. acidocaldarius* proteins.

### Construction of Chromosomally HA-Tagged *S. acidocaldarius* Mutants

To construct plasmids for chromosomally C-terminal HA-tagged mutants (Supplementary Table S1), overlap extension PCR was performed to obtain an overlap fragment containing a 500–900 bp upstream region of the original stop codon, an HA-tag encoding sequence with a new stop codon, and a 400–500 bp downstream region of the original stop codon. Overlap PCR fragments were cloned into vector pSVA407 to obtain the desired plasmids (Supplementary Table S2). After sequencing, plasmids were transformed into *E. coli* ER1821 for methylation. Methylated plasmids were electroporated into *S. acidocaldarius* MW001. Transformants were selected on 1st selection plates (0.6% gelrite) lacking uracil and subsequently grown on 2nd selection plates (0.6% gelrite) with uracil and 5-FOA (100 μg/mL). Positive mutants were screened by colony PCR with checking primers (Supplementary Table S2) and further confirmed by sequencing.

### Western Blotting

Protein samples were separated on SDS-PAGE and blotted to PVDF membrane (Roche). The membrane was incubated with primary antibody against FlaB (Eurogentec) or the HA-tag (Sigma) overnight at 4°C. Subsequently, secondary goat anti-rabbit-HRP antibody (Invitrogen) was used. His- and Strep-tagged proteins were detected directly with anti-His-HRP (α-His) (Abcam) and anti-Strep-HRP (α-Strep) (IBA) antibodies. Chemiluminescent signals were visualized by the ECL Chemocam Imager (INTAS) with the Clarity Western ECL blotting substrate (Bio-Rad). Western blots from at least three biological replicates were quantified with ImageJ (NIH).

### Motility Assays

Motility assays were performed as described (Lassak et al., 2012). Δ*arnA* and Δ*arnR/R1* strains were used as the positive and negative control, respectively.

### RNA Isolation and qRT-PCR

Total RNA samples were prepared as described (Lassak et al., 2012) from 10 mL cultures collected at indicated growth conditions and time points by the TRIzol method (Invitrogen) following the manufacturer’s instructions. QuantiTect Reverse Transcription Kit (Qiagen) was used to clean up gDNA from total RNA samples and synthesize cDNA following the manufacturer’s instructions. Quantitative PCR analysis was performed with qPCRBIO SyGreen Mix (PCR Biosystems) and Rotor-GeneQ Real-time PCR cycler (Qiagen). *Ct* values were normalized to that of the reference gene *secY*. At least three biological replicates and two technical replicates were used for analysis.

### Co-immunoprecipitation (co-IP) Assays With Anti-HA-Magnetic Beads

For each co-IP assay, 500 mL of *S. acidocaldarius* culture was used, which was collected after 0.5 h growth in nutrient starvation medium (Brock’ basal medium (pH 3.0–3.5) supplemented with 10 μg/mL uracil) at 75°C and 120 rpm. The culture was cooled down on ice until the temperature approached around 37°C, and cells were collected by centrifugation, then re-suspended in lysis buffer (25 mM Tris, pH 7.4, 150 mM NaCl, 1 mM EDTA, 1% NP40, 5% glycerol) supplemented with protease inhibitor and DNase I, and disrupted by French press (Thermo). Cell debris was removed by centrifugation at 48, 000 × *g* at 4°C for 20 min and at 236,000 × *g* and 4°C for 45 min. After centrifugation, the supernatant was used for co-IP assays with Pierce HA-tag IP/co-IP kit (Pierce) as described in manufacturer’s instructions. 20 μL of the elution fraction was used for SDS-PAGE. The proteins in the co-IP elution fractions were analyzed by silver staining and mass spectrometry.

### LC-MS/MS Analysis

Mass spectrometric analysis was basically carried out as described in Schwenk et al. (2014). Briefly, peptides obtained from tryptic in-gel digests were dissolved in 0.5% trifluoroacetic acid and aliquots were loaded onto a C18 PepMap100 precolumn (particle size 5 mm; Dionex/Thermo Scientific, Germany) with 0.5% (v/v) acetic acid using a split-based UltiMate 3000 HPLC (Dionex/Thermo Scientific, Germany). Bound peptides were eluted and separated with an aqueous-organic gradient from 0.5% (v/v) acetic acid to 0.5% (v/v) acetic acid in 80% (v/v) acetonitrile (1 h 20′ total) in a PicoTip emitter (i.d. 75 mm; tip 8 mm; New Objective, United States) manually packed with ReproSil-Pur 120 ODS-3 (C18; particle size 3 mm; Dr. Maisch HPLC, Germany) and electrosprayed (2.3 kV; transfer capillary temperature 250°C) into an LTQ Orbitrap XL tandem mass spectrometer with the described settings.

### Protein Identification

Peak lists were extracted from fragment ion spectra using the “msconvert.exe” tool (part of ProteoWizard, v3.0.6906, proteowizard.sourceforge.net; Mascot generic format with filter options “peakPicking true 1-” and “threshold count 500 most-intense”) and the precursor *m*/*z* values were shifted by the median *m*/*z* offset of all peptides assigned to proteins in a preliminary database search with 15 ppm. Corrected peak lists were searched with Mascot 2.6 (Matrix Science, United Kingdom) against a *Sulfolobus acidocaldarius* proteome database (UniProtKB/Swiss-Prot UP000001018 release 2019-11, supplemented with entries for common exogenous contaminations).

Acetyl (Protein N-term), Carbamidomethyl (C), Ethyl (K), Formyl (N-term), Formyl (S), Formyl (T), Gln→pyro-Glu (N-term Q), Glu→pyro-Glu (N-term E), Oxidation (M) and up to one missed tryptic cleavage were allowed as variable modifications. Peptide and fragment mass tolerance were set to ±5 ppm and ±0.8 Da, respectively. The expect value cut-off for peptide assignment was set to 0.5. Proteins either representing exogenous contaminations (e.g., keratins, trypsin, IgG chains) or identified by only one specific peptide were not considered.

### Protein Quantification

Label-free quantification of proteins was carried out as described in (Schmidt et al., 2017). Peptide signal intensities (peak volumes, PVs) from MS full scans were determined and offline mass calibrated using MaxQuant v1.6.3.3^1^. Peptide PV elution times in evaluated datasets were pairwise aligned using LOESS regression (reference times were dynamically calculated from the median peptide elution times over all aligned datasets). PVs were then assigned to peptides based on their *m*/*z* and elution time obtained either directly from MS/MS-based identification or inferred indirectly using in-house developed software (matching tolerances of ±2 ppm and ±1 min). Molecular abundances of proteins were estimated using the abundance_norm_spec score defined as the sum of all assigned and protein isoform-specific PVs divided by the number of MS-accessible protein isoform-specific amino acids (Bildl et al., 2012). Co-purification of proteins was evaluated by calculating protein *ratios* from their 6 or 50% (whatever number is higher) most consistent peptide PVs [TopCorr method, (Bildl et al., 2012)] in the respective target versus control APs.

### Overexpression and Purification of Recombinant Proteins in *E. coli*

20 mL of the overnight culture was inoculated into 2 L LB medium with corresponding antibiotics. Cells were grown at 37°C until they reached an OD_600_ of 0.5–0.6 and induced with isopropyl β-D-1-thiogalactopyranoside (IPTG) (final concentration 500 μM). Subsequently, growth was continued overnight at 16°C. Cells were collected by centrifugation, suspended in lysis buffer (50 mM Tris, pH 8, 150 mM NaCl) supplemented with protease inhibitor and DNase I and disrupted by the French press (Thermo). Cell debris and inclusion bodies were removed by centrifugation at 48,000 *g* at 4°C for 20 min. A heat shock step was performed with the supernatant at 70°C for 20 min, followed by ultracentrifugation at 236,000 *g* for 45 min. The supernatant was used for affinity purification. His-tagged proteins (ArnA, ArnB, PP2A, and ArnC) were purified using Ni-NTA beads. Purification was performed in lysis buffer using a 10–200 mM imidazole gradient on a gravity column. Strep II-tagged proteins (ArnA and ArnB) were purified using streptavidin beads. A concentration gradient of 2.5 mM desthiobiotin in lysis buffer, was used to elute Strep II-tagged proteins from the column. Elution fractions from affinity purification were applied to a Superdex 200 (16/600) size exclusion column (SEC; GE Healthcare Life Sciences) which was pre-equilibrated with lysis buffer.

### Protein Complex Reconstitution

To reconstitute the ArnA/B complex *in vitro*, equal amounts of purified ArnA and ArnB were mixed and incubated on ice for 1 h. After incubation, the mixture was applied to a Superdex 200 (16/600) size exclusion column to separate the reconstituted complex from the individual proteins. The size exchange chromatography fractions were analyzed by SDS-PAGE and Coomassie blue staining. The fractions containing the reconstituted complex were pooled for further use.

### *In vitro* Phosphorylation Assays

*In vitro* phosphorylation assays were performed as described (Reimann et al., 2012) at 55°C in the presence and the absence of the kinase ArnC (100 nM) and [γ-^32^P] ATP (32 nM) in lysis buffer containing 1 mM MnCl_2_ and 2 μM protein (ArnA, ArnB, or ArnA/B complex).

### *In vitro* Dephosphorylation Assays

To prepare ^32^P-labeled phosphorylated protein samples, *in vitro* phosphorylation assays were performed with 100 nM kinase ArnC and 32 nM [γ-^32^P] ATP in lysis buffer containing 1 mM MnCl_2_ and 2 μM protein (ArnA, ArnB, or ArnA/B complex). After 40 min incubation at 55°C, 100 fold excess of non-labeled ATP and PP2A (200 nM) were added and samples were taken at different time points.

### *In vitro* Protein-Protein Interaction Analysis

*In vitro* protein-protein interaction analysis was performed with purified His- and Strep-tagged proteins in buffer containing lysis buffer. 500 μL of each protein (0.3 mg/mL) was mixed and incubated at 55°C for 30 min. The mixture was applied to a 1000 μL PureSpeed tip with 20 μL IMAC resin (Mettler Toledo) according to the manufacturer’s instruction. For the elution step, 50 μL of lysis buffer containing 500 mM imidazole was used. All collected fractions were analyzed by Western blotting using α-His and α-Strep II antibodies. To obtain phosphorylated Strep-tagged proteins, the respective protein was phosphorylated by addition of 8 μg His-tagged ArnC and incubation at 55°C for 30 min. His-tagged ArnC was removed by passing the sample over a PureSpeed Tip, and the flowthrough fraction was phosphorylated Strep-tagged protein sample, which was used for the interaction analysis with His-tagged proteins.

### Analytical Size Exclusion Chromatography

Analytical size exclusion chromatography was performed on either a Superdex 200 (16/600) or a Superdex 200 increase (10/300) size exclusion column, run in lysis buffer at 1.0 and 0.7 mL/min. To analyze the effect of phosphorylation and dephosphorylation on the stability of the ArnA/B complex, 300 μg of purified ArnA/B complex were incubated with either 8 μg ArnC or 40 μg of PP2A. The injection volumes were 2000 and 500 μL, respectively, and the following protein standards (Bio-Rad) were used: Thyroglobulin (bovine) 670,000 Da, γ-globulin (bovine) 158,000 Da, Ovalbumin (chicken) 44,000 Da, Myoglobin (horse) 17,000 Da, Vitamin B12 1,350 Da.

### Mass Photometry

Protein samples were diluted from 10x stocks to end concentrations of 20–100 nM in 50 mM Tris-buffer at pH 8.0 and 150 mM NaCl. The measurements were performed on a Refeyn OneMP using cleaned High Precision Microscope Cover Glasses (24 × 60 mm, Marienfeld) and silicon gaskets (CultureWell^TM^ reusable gaskets, SKU: 103250). The gaskets were filled with 18 μL buffer to adjust the focus using the maximum sharpness level between 6 and 7. Then, 2 μL of the protein solution were added and briefly mixed before starting the acquisition of 6000–12000 images. To convert the obtained contrast values to molar masses, a standard curve was prepared prior to the experiment in the same buffer as described above using known masses from the Novex^TM^ NativeMARK^TM^ protein standard (Invitrogen). All solutions and buffers were sterile-filtered prior to use. The histograms were processed and fitted with the Refeyn Discover^MP^ analysis software (v2.2.0).

## Results

PTP and PP2A are the only two protein phosphatases in *S. acidocaldarius*. Deletion mutants of these phosphatases show higher phosphorylation levels of several proteins in the archaellum regulatory network and deletion of PP2A resulted in increased expression of the archaellum and increased motility (Reimann et al., 2013). However, the details on how they regulate this process are not clear. Systematic analysis of protein-protein interactions has been an efficient approach to unravel a complicated interaction network (Breitkreutz et al., 2010). In this study, we set out to identify the proteins in the archaellum regulatory network that interacted with the phosphatases via co-IP.

### Expression of HA-Tagged PTP and PP2A in *S. acidocaldarius*

**FIGURE 1:**
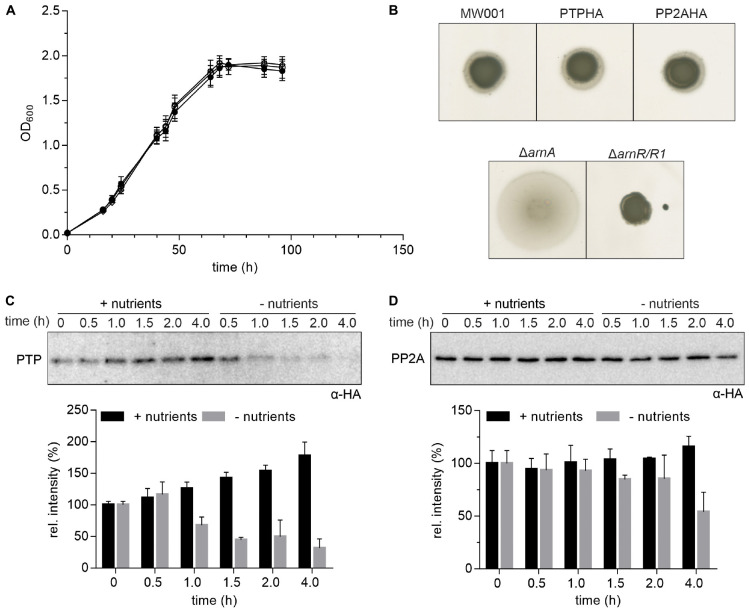
Expression of HA-tagged PP2A and PTP. **(A)** Growth curves of *S. acidocaldarius* cells grown in nutrient rich medium at 75°C were obtained for the parental MW001 strain (black solid circle) and the stains containing HA-tagged PP2A (black open square) and PTP (black open circle). The OD_600_ of each strain was measured at the indicated time points. The average of three independent experiments is shown. **(B)** For motility assays, exponentially growing *S. acidocaldarius* cultures of around OD_600_ 0.4 were spotted on semi-solid gelrite plates with 0.005% (w/v) NZ-amine and incubated at 75°C for 5 days before scanning the plates. The motility assays were repeated with three biological and six technical replicates. A representative experiment is shown. **(C)** Expression of HA-tagged PTP and **(D)** PP2A in *S. acidocaldarius*. PTPHA and PP2AHA mutants were grown in nutrient rich and starvation medium for 4 h. Samples were collected at different time points (0, 0.5, 1.0, 1.5, 2.0, and 4.0 h) and analyzed by Western blotting with α-HA. Representative Western blots are shown. A quantification of three independent experiments is shown below the blots.

Sequences encoding C-terminal HA-tags were integrated into the genome to facilitate the co-IP of PTP and PP2A. To exclude functional defects or any polar effects of PTP and PP2A in *S. acidocaldarius* due to the insertion of the HA-tag, motility tests on semi-solid gelrite plates and growth under nutrient rich conditions of both mutants were compared to the parental MW001 strain. Neither for the swimming behavior nor for the general growth, differences could be observed (Figures 1A,B). These results indicate that the insertion of the C-terminal HA-tags did not affect the function of either PTP or PP2A *in vivo*.

Previously, 4 h after transfer to starvation medium, archaella could be detected by electron microscopy and swimming cells could be observed using the thermo-microscope (Haurat et al., 2017). To determine the best time point to perform the co-IP, the expression levels of PTP, PP2A and the archaellin FlaB, which encodes the filament protein of the archaellum, were determined at different time points during 4 h of growth in nutrient rich and starvation medium (Supplementary Figure S1). Expression of PTP increased over 4 h of growth in nutrient rich medium while it decreased significantly from 1.0 h of growth in nutrient starvation medium (Figure 1C). Expression of PP2A was higher than expression of PTP and did not change significantly during growth in both media except for a slight decrease after 4.0 h of growth in starvation medium (Figure 1D).

The expression of FlaB was determined both on mRNA transcript level as well as on protein level. After 0.5 h, an initial increase in the transcript levels of *flaB* was detected followed by an increase of FlaB protein levels after 1–1.5 h (Supplementary Figure S2). Since it was expected that interactions of PTP and PP2A with the components of the archaellum regulatory network might occur especially during the earlier phases of archaellum expression, samples for co-immunoprecipitation were collected 0.5 h after transfer to starvation medium.

Localization studies of PTP and PP2A in cells grown in starvation medium for 0.5 h showed that PTP and PP2A could mainly be identified in the cytoplasmic fractions and not in the isolated membranes (Supplementary Figure S3). Therefore, the co-IPs were performed without the addition of detergents to solubilize possible membrane components.

### Identification of Proteins Associated With PTP and PP2A

To identify proteins associated with PTP and PP2A, *S. acidocaldarius* cells were collected after 0.5 h growth in starvation medium. Cytoplasmic fractions of cell samples were prepared and used for co-IP assays with anti-HA magnetic beads. The parental MW001 strain was used as a standard. To increase specificity, the co-IP was performed without addition of cross-linkers. Proteins in elution fractions were separated on SDS-PAGE and visualized by silver staining (Supplementary Figure S4).

The PTP and PP2A bands could clearly be observed in PTPHA and PP2AHA samples, respectively. As expected, the intensity of PP2A was higher than that of PTP. No significant visible difference could be observed between the protein profiles of MW001 and PTPHA, but some additional bands were observed when PP2AHA was compared with MW001.

**FIGURE 2:**
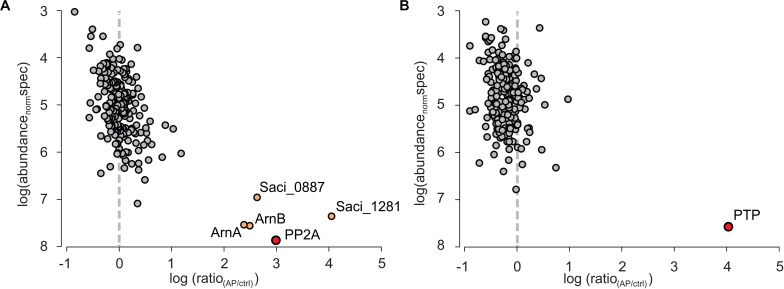
Quantitative mass spectrometric analysis of PP2A and PTP affinity purifications (APs) from *S. acidocaldarius*. Individual proteins identified and quantified are displayed as circles in logarithmic plots of their molecular abundance (calculated as abundance_norm_ spec values, see Methods) versus their enrichment (ratio) in the target AP for **(A)** HA-PP2A and **(B)** HA-PTP relative to a control AP from wt cells. Circles in gray indicate non-specifically binding proteins, whereas the primary AP target proteins and specifically co-purified interactors are colored in red and light orange, respectively.

The co-IP samples were analyzed by mass spectrometry and molecular abundance of the identified proteins was plotted versus their enrichment compared to the co-IPs performed in MW001 (Figure 2). For PTPHA, no highly enriched interaction proteins were identified, but for PP2AHA a conserved putative ATP/GTP binding protein (Saci_1281), a universal stress protein (Saci_0887) and the archaellum regulators ArnA and ArnB were identified as highly enriched after PP2A-HA co-IPs. Homologs of both the putative ATP/GTP binding protein Saci_1281 and the universal stress protein Saci_0887 are highly conserved and are involved in many different processes (see discussion). The negative regulators for archaellum expression ArnA and ArnB strongly interact with each other both *in vitro* and *in vivo*, and their deletion resulted in a significant increase of archaellum gene expression (Reimann et al., 2012). Their expression is kept constant in cells, however, their interaction is sequentially abolished in nutrient starved cells (Hoffmann et al., 2019). The C-terminal domain of ArnB could be phosphorylated by ArnC *in vitro* (Hoffmann et al., 2017) and was found phosphorylated *in vivo*, which suggested that phosphorylation/dephosphorylation of this domain might influence its interaction with ArnA (Hoffmann et al., 2019). Indeed, here ArnA and ArnB are identified as highly abundant interaction proteins of PP2A. Therefore, the interaction between ArnA, ArnB and the phosphatase PP2A is studied in more detail.

### ArnA and ArnB Form a Stable Complex *in vitro*

**FIGURE 3:**
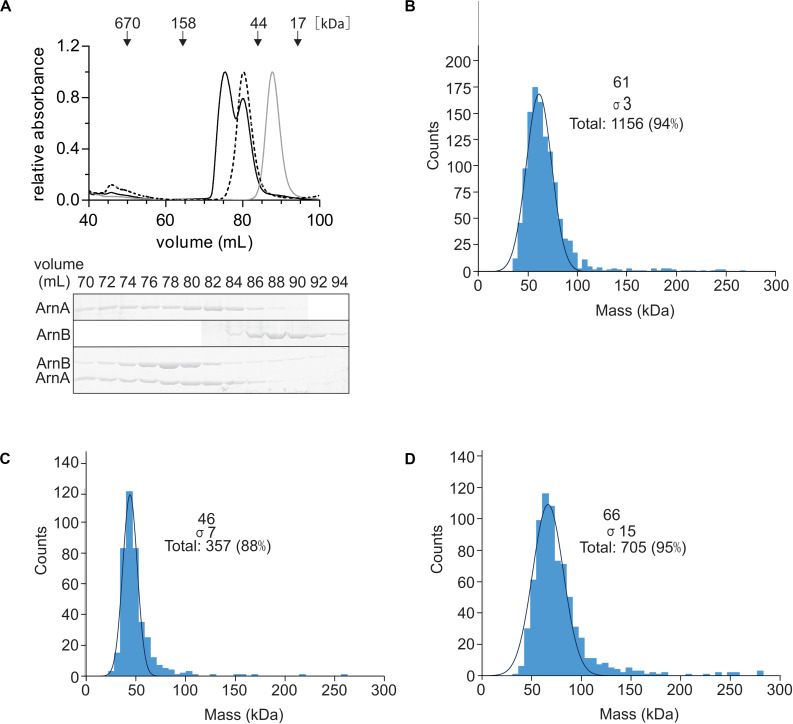
Reconstitution of the ArnA/B complex *in vitro*. **(A)** Purified Strep-ArnA (dashed line), ArnB-His (gray line), or 300 μg of ArnA-His and ArnB-Strep (black line) were incubated on ice for 1 h were loaded on a SD200 16/600 size exclusion column. Elution fractions were separated on SDS-PAGE and analyzed by Coomassie staining. The SDS-PAGE of the protein fractions eluting from the size exclusion column for the experiment where equal amounts ArnA-His and ArnB-Strep were mixed is shown. Mass photometry of **(B)** 100 nM ArnA, **(C)** 20 nM ArnB and **(D)** 50 nM ArnA/B complex.

To test whether the ArnA/B complex could be reconstituted from the purified proteins, N-terminally Strep-tagged ArnA and C-terminally His-tagged ArnB were overexpressed and purified from *E. coli*. After size exclusion chromatography, Strep-ArnA and ArnB-His, eluted as symmetric peaks at volumes corresponding to molecular masses of ∼60 and 40 kDa, respectively (Figure 3A). When Strep-ArnA and ArnB-His were mixed, they eluted at a higher molecular mass of ∼80 kDa compared to the individual proteins, indicating that ArnA and ArnB formed a stable complex *in vitro*. Especially the shift in the elution pattern of ArnB was apparent. To confirm this, using a different method, 300 μg of ArnA-His, ArnB-His and the fractions containing the ArnA-His/ArnB-His complex were again loaded on a size exclusion chromatography and elution fractions containing ArnA, ArnB, and the ArnA/B complex were analyzed using mass photometry (Figures 3B–D) which resulted in molecular masses of 63.7 ± 2.3, 47.0 ± 1.7, and 66.0 ± 1.0 kDa, respectively. Combining the results of the gel filtration and the mass photometry experiments with the molecular masses of Strep-ArnA and ArnB-His of 26.8 kDa and 43.6, it is concluded that ArnA forms a dimer and that most likely ArnA and ArnB form a complex with a 1:1 stoichimetry.

### Protein Phosphorylation Stimulates Further Complex Formation of ArnA and ArnB

**FIGURE 4:**
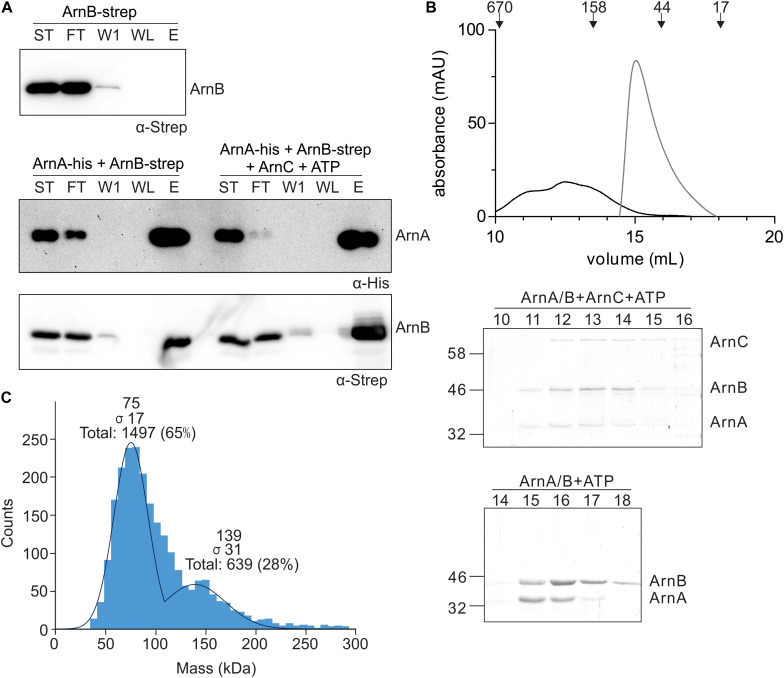
*In vitro* interaction analysis assays of ArnA and ArnB. **(A)** ArnB-Strep (upper panel), ArnA-His and non-phos ArnB-Strep (left lanes of middle and lower panel) or ArnA-His and phos-ArnB-Strep (right lanes of middle and lower panel) were incubated at 55°C for 30 min and applied to a Ni-affinity resin, washed and eluted with imidazole. Fractions were analyzed by Western blotting with α-His (middle panel) and α-Strep antibodies (upper and lower panel). ST: starting materials; FT: flow-through; W1: 1st wash fraction; WL: last wash fraction; E: elution fraction. **(B)** ArnA/B complex collected from Figure 3A were incubated with Mn^2+^ and ATP in the presence (black line) and absence (gray line) of ArnC at 55°C for 30 min and were loaded on a Superdex 200 increase (10/300) size exclusion column. The upper panel depicts the elution pattern observed at 280 nm. Elution fractions were separated on SDS-PAGE and analyzed by Coomassie staining. The SDS-PAGEs of the protein fractions around the peaks observed are shown in the lower panel. **(C)** Mass photometry of the 50 nM ArnA/B complex.

To study the effect of protein phosphorylation on the ArnA/B interaction, *in vitro* protein interaction assays between ArnA-His and ArnB-Strep were performed with non-phosphorylated and phosphorylated ArnB-Strep. To obtain phosphorylated ArnB, ArnB was incubated with ArnC and ATP in the presence of Mn^2+^. Samples were loaded on a Ni-NTA resin, washed, and eluted with imidazole, and the samples were loaded on gel (Figure 4A). ArnB-Strep was retained on the column in the presence of ArnA-His, but was not retained in the absence of ArnA-His, again demonstrating the interaction between ArnA and ArnB *in vitro.* Remarkably, significantly more ArnB-Strep was retained on the column when ArnB-Strep was phosphorylated, which shows that protein phosphorylation increases the ArnA-ArnB interaction. To analyze a possible effect of protein phosphorylation on the oligomeric state or stoichiometry of the ArnA/B complex, the phosphorylated ArnA/B complex was analyzed by size exclusion chromatography (Figure 4B) and mass photometry (Figure 4C). Phosphorylation of the ArnA/B complex led to the formation of complexes, which eluted at elution volumes corresponding to a molecular mass of 60-540 kDa. Remarkably, phosphorylation of ArnA also led to an upshift of ArnB to a higher molecular mass. Mass photometry was performed using the phosphorylated ArnA/B complex and showed concentration dependent formation of an additional ArnA/B complex with a mass of 136.0 ± 8.9, which most likely represents an (ArnA/B)_2_ tetrameric complex. This was dependent on the Mn^2+^ dependent phosphorylation of the complex by ArnC, since no formation of (ArnA/B)_2_ or higher oligomeric complexes were observed in the presence of only Mn^2+^ or ArnC in the presence of the Mn^2+^ chelator EDTA (see Supplementary Figure S5).

### ArnA/B Complex Formation Stimulates Phosphorylation of ArnB by ArnC and Dephosphorylation of ArnB by PP2A

**FIGURE 5:**
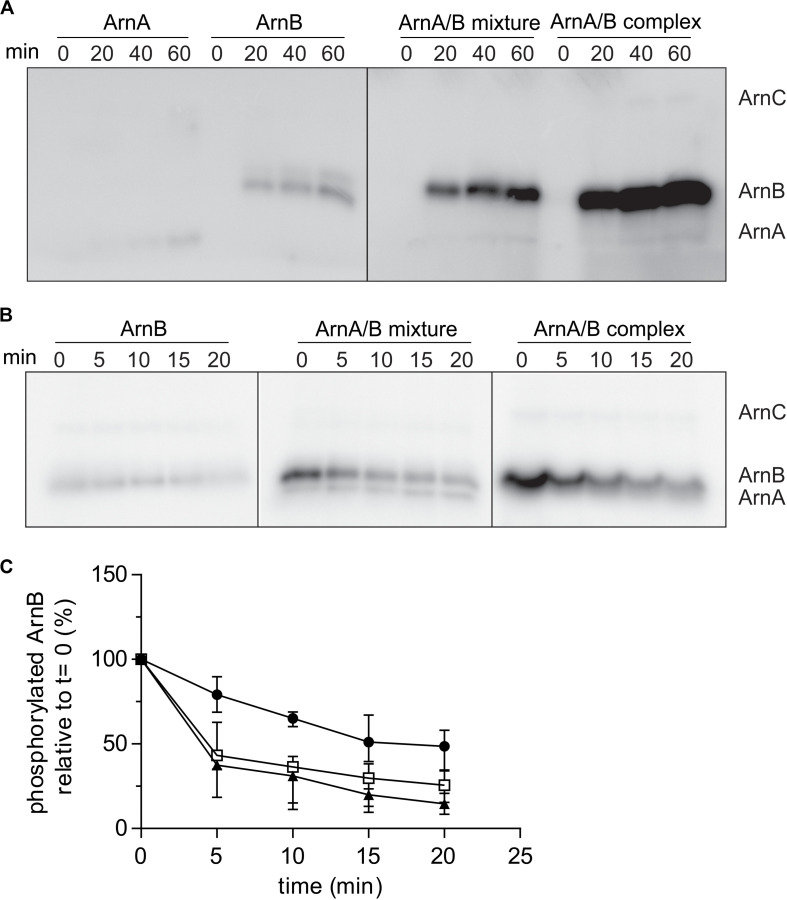
Rates of phosphorylation of ArnB by ArnC and dephosphorylation by PP2A are increased in the ArnA/B complex. **(A)** 2 μM ArnA, ArnB, mixed ArnA/B mixture or isolated ArnA/B complex were incubated at 55°C in the presence of 100 μM ATP supplemented with [γ−^32^P] ATP and 100 nM ArnC for different times, separated on SDS-PAGE and the phosphorylation levels were determined by phosphoimaging. **(B)** 2 μM ArnB, mixed ArnA/B or the isolated ArnA/B complex were first incubated at 55°C in the presence of 100 μM ATP supplemented with [γ−^32^P] ATP and 100 nM ArnC. After 40 min, 100 fold excess of non-labeled ATP and 200 nM PP2A were added and samples were taken at different time points, separated on SDS-PAGE and the gels were analyzed by phosphoimaging (upper panel). **(C)** Quantification of phos ArnB signals from three independent dephosphorylation assays. Black circles, ArnB; empty squares, ArnA/B mixture; black filled triangles, ArnA/B complex.

To test whether ArnA/B complex formation affected the phosphorylation kinetics of ArnA and ArnB, the phosphorylation kinetics of Strep-ArnA, ArnB-His, a mixture of Strep-ArnA and ArnB-His and the isolated ArnA/B complex by ArnC were determined (Figure 5A). As seen previously, ArnB was phosphorylated at a higher rate than ArnA, but much higher phosphorylation rates and final phosphorylation levels were seen for ArnB in the Strep-ArnA/ArnB-His mixture and even more pronounce for the co-purified ArnA/B complex. This suggests that formation of the ArnA/B complex results in a conformational change in ArnB, which makes the phosphorylation sites in ArnB more accessible to ArnC. To investigate whether PP2A can dephosphorylate ArnB, dephosphorylation assays with PP2A were performed (Figure 5B). ArnB-His, a mixture of Strep-ArnA and ArnB-His and the isolated ArnA/B complex were first phosphorylated by ArnC using ^32^P-γ-ATP after which an excess of non-labeled ATP (to prevent further phosphorylation with ^32^P) and PP2A were added (Figure 5B). As expected, purified PP2A can dephosphorylate both free ArnB and ArnB in complex with ArnA, but not ArnA itself. The phosphorylation signal of ArnB was quantified and the dephosphorylation kinetics were plotted (Figure 5C). This showed that dephosphorylation of ArnB in complex with ArnA occurred with a higher rate than dephosphorylation of free ArnB. Thus, both phosphorylation by ArnC and dephosphorylation of ArnB by PP2A are stimulated in the ArnA/B complex (Figures 5A,C).

### PP2A Interacts Strongest With Phosphorylated ArnB and Dephosphorylation of ArnB Results in Complex Dissociation

**FIGURE 6:**
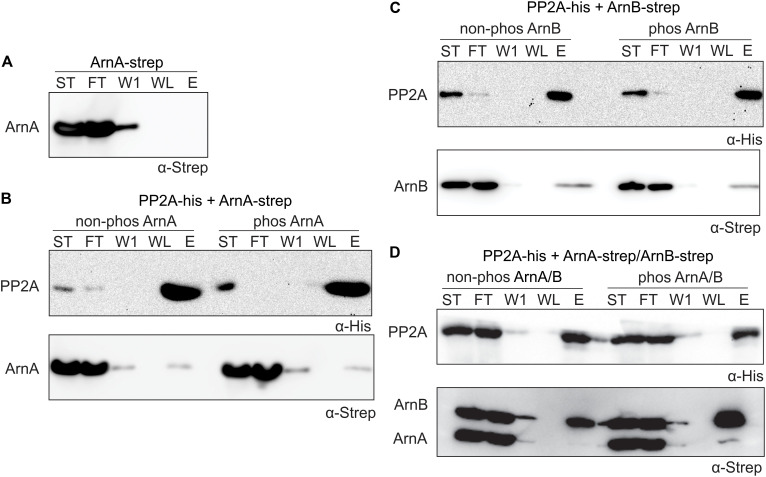
PP2A interacts stronger with the phosphorylated ArnA/ArnB complex and dephosphorylation of the complex results in complex dissociation. **(A)** Strep-ArnA, **(B)** PP2A-his and non-phos/phos Strep-ArnA, **(C)** PP2A-his and non-phos/phos ArnB-Strep, and **(D)** PP2A-his and non-phos/phos Strep-ArnA/ArnB-Strep complex, were incubated at 55°C for 30 min and were applied to a Ni-affinity resin, and washed and eluted with imidazole. Fractions were analyzed by Western blotting with α-His (upper panel) and α-Strep antibodies (upper and lower panel). ST, starting materials; FT, flow-through; W1, 1st wash fraction; WL, last wash fraction; E, elution fraction.

To determine whether ArnA/B complex formation and phosphorylation influences the interaction with PP2A, *in vitro* protein interaction assays between PP2A-His and Strep-ArnA and ArnB-Strep and the Strep-ArnA/ArnB-Strep complex with and without phosphorylation by ArnC were performed. ArnB-Strep (Figure 4) and Strep-ArnA (Figure 6A) were not retained on the column in the absence of PP2A-His. Small amounts of Strep-ArnA (Figure 6B) and ArnB-Strep (Figure 6C) were retained on the column when they were incubated with PP2A-His, and no significant differences were observed when Strep-ArnA or ArnB-Strep were phosphorylated by ArnC. Most likely no effects were observed here due to the relatively low phosphorylation rates of the single ArnA and ArnB proteins (see Figure 5). Much more ArnB-Strep was retained on the column when PP2A-His was incubated with the Strep-ArnA/ArnB-Strep complex and the amount of ArnB-Strep retained on the column was increased after phosphorylation of the ArnA/B complex by ArnC (Figure 6D). This shows that PP2A interacts with higher affinity with phosphorylated ArnB in the presence of ArnA. Remarkably, this interaction seems to result in destabilization of the ArnA/B complex, since almost no co-purified ArnA was found.

**FIGURE 7:**
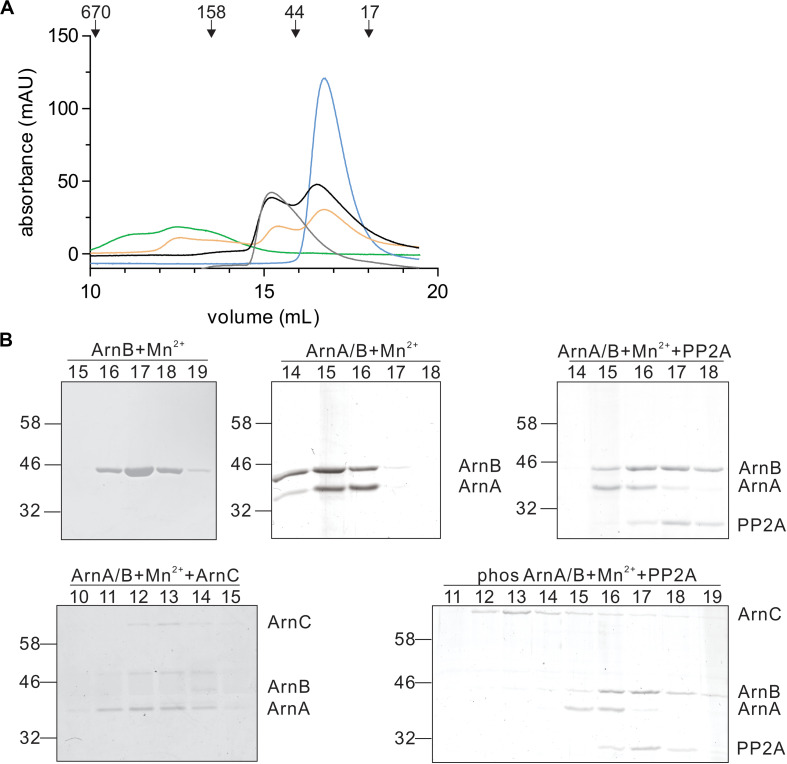
Interaction with PP2A and dephosphorylation dissociates the ArnA/B complex. **(A)** Samples were incubated with Mn^2+^ at 55°C and were loaded on a Superdex 200 increase (10/300) size exclusion column. ArnB (blue line) or the ArnA/B complex (gray line) were incubated for 30 min. The ArnA/B complex was incubated for 30 min in the presence (orange line) or absence (black line) of ArnC and ATP followed by incubation with PP2A for 30 min. The ArnA/B complex incubated for 30 min with ArnC and ATP was indicated as a green line. The elution patterns observed at 280 nm are shown. **(B)** Elution fractions were separated on SDS-PAGE and analyzed by Coomassie staining. The SDS-PAGE of the protein fractions around the peaks observed are shown.

**FIGURE 8:**
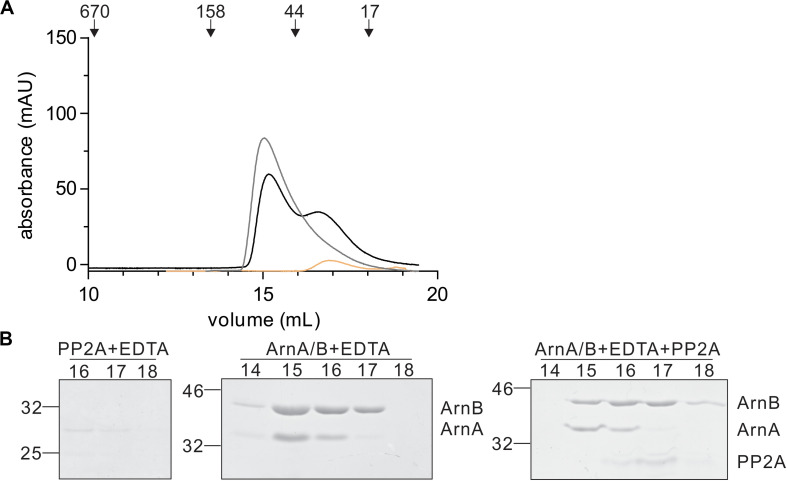
Binding of PP2A to the ArnA/B complex suffices to dissociate the complex. **(A)** PP2A (orange line) or the ArnA/B complex (gray line) were incubated for 30 min with EDTA at 55°C. The ArnA/B complex was incubated for 30 min in the presence of EDTA (black line) at 55°C followed by incubation with PP2A for 30 min at 55°C. Samples were loaded on a Superdex 200 increase (10/300) size exclusion column. The elution patterns observed at 280 nm are depicted. **(B)** Elution fractions were separated on SDS-PAGE and analyzed by Coomassie staining. The SDS-PAGE of the protein fractions around the peaks observed are shown.

In a final step, the effect of interaction with PP2A and dephosphorylation on the ArnA/B complex was analyzed by size exclusion chromatography (Figures 7A,B). Incubation of the ArnA/B complex and the phosphorylated oligomeric ArnA/B complex with PP2A in the presence of Mn^2+^ resulted in the dissociation of the complexes to ArnA and ArnB, demonstrating that interaction with PP2A and dephosphorylation reverses (ArnA/B)_n_ complex formation. Thus, phosphorylation by ArnC results in formation of (ArnA/B)_2_ and higher oligomeric complexes, while interaction and dephosphorylation by PP2A results in dissociation of these complexes. Since PP2A is a Mn^2+^ dependent phosphatase, addition of the Mn^2+^ chelator EDTA allowed to test whether the interaction with PP2A suffices to dissociate the ArnA/B complex in the absence of phosphatase activity (Figure 7). Indeed the ArnA/B complex dissociated after the incubation with EDTA and PP2A (Figure 8), indicating that interaction of PP2A with the ArnA/B complex is sufficient to dissociate the ArnA/B complex. It, however, seems likely that the phosphatase activity of PP2A would further contribute to the phosphorylated oligomeric ArnA/B complex dissociation. Importantly, addition of EDTA to the ArnA/B complex did not affect the oligomeric state of the ArnA/B complex (Figure 8), demonstrating that the metal binding MIDAS site of ArnB is not involved in complex formation.

## Discussion

Here we set-out to study the role of the PTP and PP2A phosphatases of *S. acidocaldarius*, especially focusing on their roles in the synthesis of the archaellum in response to nutrient starvation. Fusion of PTP and PP2A with C-terminal HA-tags did neither affect growth rates nor swimming motility *in vivo* and allowed the determination of the levels of PTP and PP2A during normal growth and during growth on starvation medium. Expression of PP2A did not change significantly during growth on rich medium or in the first four hours after transfer to starvation medium, while the levels of PTP increased during growth in nutrient rich medium while they decreased during growth in nutrient starvation medium. Thus, only the levels of PTP, but not those of PP2A, change under starvation conditions. Remarkably, only deletion of the gene encoding PP2A, but not the gene encoding PTP affected motility, demonstrating a more important role for PP2A than PTP in the regulation of motility. Our observation that the levels of PP2A did not change during growth, indicate that the effects of protein phosphorylation levels on motility are regulated by changing the expression/activity of protein kinases like Saci_0965, ArnC, ArnD, and ArnS (Haurat et al., 2017; Hoffmann et al., 2017) but that possibly also other factors might have an effect on the phosphatase activity of PP2A.

Since it was expected that interactions of the phosphatases with the components of the archaellum regulatory network or with proteins that change the activity of the phosphatases occur during the earlier phases of archaellum expression, samples for co-immunoprecipitation (co-IP) were collected 0.5 h after transfer to starvation medium. At this point, the first expression of FlaB was detected and both PP2A and PTP are expressed. Since many Ser/Thr phosphorylated or Tyr-phosphorylated proteins were previously identified (Reimann et al., 2013) and only two phosphatases are present in *S. acidocaldarius*, many interaction partners were expected and, to identify the most significant partners, co-IP experiments were performed without cross-linking and under stringent conditions. For PTP-HA, under these conditions no highly enriched interaction proteins were identified, but for PP2A-HA, Saci_0887, a universal stress protein (USP), Saci_1281, a conserved putative ATP/GTP binding protein, and the archaellum regulators ArnA and ArnB were highly enriched after co-IPs. The high specificity of these interactions suggests that next to possible phosphorylated substrates for PP2A these proteins might be interacting partners that modulate the activity of PP2A.

Saci_1281, a GPN-loop GTPase was the highest enriched protein identified in the PP2A pull-down. Small GTPases are widely distributed in all three domains of life and function as molecular switches cycling between GTP-bound and GDP-bound states which modulate downstream effector proteins which are involved in a variety of cellular processes (Wittinghofer and Vetter, 2011). GPN loop GTPases contain a conserved Gly-Pro-Asn motif (named GPN loop) and have only been identified in Archaea and Eukarya. In Eukarya, often three homologs (GPN1, GPN2, and GPN3) are found, which are involved in nuclear localization and biogenesis of RNAPII (Wittinghofer and Vetter, 2011; Alonso et al., 2013; Niesser et al., 2016; Guerrero-Serrano et al., 2017). In Archaea, generally one homolog is encoded, but the physiological function is not known yet. The GPN-loop GTPase PAB0955 from *Pyrococcus abyssi* was found to interact with DNA topo-isomerase VI (subunit B), DNA primase DnaG and RF-C (small subunit) and was able to autophosphorylate on Ser and Thr residues (Gras et al., 2007). In a phosphoproteome study of *S. acidocaldarius*, Saci_1281 was found phosphorylated at Tyr-59, but no Ser or Thr phosphorylation was found (Reimann et al., 2013).

The second most enriched protein identified in the PP2A pull-down was the universal stress protein Saci_0887. Members of the universal stress protein (USP) superfamily have been identified in all three kingdoms of life (Vollmer and Bark, 2018). Many bacterial species have multiple paralogs of genes encoding these proteins and deletion of the genes often does not provide a distinct phenotype. Generally, USP proteins are expressed under different environmental stress conditions, which suggests an important role of these proteins in general stress responses (Kvint et al., 2003; Vollmer and Bark, 2018). In bacteria, USP proteins were found to be involved in cell growth and survival under stress conditions, motility, biofilm formation, and virulence of pathogen to host (Kvint et al., 2003; Nachin et al., 2005; O’Connor and McClean, 2017; Vollmer and Bark, 2018). In plants, these proteins were critical for the tolerance against abiotic stresses including drought, osmotic stress and anoxia (Vollmer and Bark, 2018). Although the physiological function and structural diversity of USPs has been extensively investigated, the mechanisms by which USPs function are still largely unknown. Remarkably, several USP proteins have been shown to be phosphorylated (Freestone et al., 1997), but in a phosphoproteome study of *S. acidocaldarius*, Saci_0887 was not identified as a phosphorylated protein (Reimann et al., 2013). To our knowledge this is the first time a strong interaction between an USP and a phosphatase is identified.

The universal stress protein Saci_0887 and the GTPase Saci_1281 are likely candidates to modulate the activity of the PP2A phosphatase which might regulate different cellular processes, and this modulation and regulation should be the subject of further studies. Here we focused on the biochemical characterization of the interaction between PP2A and ArnA and ArnB, two members of the archaellum regulatory network.

ArnA and ArnB were previously shown to interact and to be present at constant levels even under nutrient limitation (Hoffmann et al., 2019). Remarkably, the strong interaction between ArnA and ArnB, which was observed during growth on rich medium, was sequentially diminished after transfer to starvation medium (Hoffmann et al., 2019). Since phosphorylated forms of ArnA and ArnB have been detected *in vivo* and *in vitro* (Reimann et al., 2012; Hoffmann et al., 2019), it was hypothesized that the interaction between ArnA and ArnB depends on their phosphorylation state. Here we have shown that the 1:1 stoichiometric ArnA/B complex can be reconstituted from purified recombinant components. Remarkably, a strong feed-back loop was observed where increased phosphorylation by ArnC resulted in more ArnA/B complex formation, which strongly stimulated phosphorylation of ArnB by ArnC, resulting in a highly phosphorylated ArnB in an oligomeric ArnA/B complex. PP2A then interacted strongly to this phosphorylated ArnB resulting in dephosphorylation of ArnB and complex dissociation.

Formation and dissociation of the ArnA/B complex is based on a delicate equilibrium of complex formation/phosphorylation by kinases and interaction with the PP2A phosphatase/dephosphorylation and complex dissociation. Analysis of this regulatory network is further complicated by the direct, indirect and possibly overlapping effects the kinases and the phosphatase have on other phosphorylated proteins in the archaellum regulatory network like the ArnR, ArnR1, and AbfR1 regulators and their binding to DNA. These interactions should be targeted by future studies.

## Data Availability Statement

The original contributions presented in the study are included in the article/Supplementary Material, further inquiries can be directed to the corresponding author.

## Author Contributions

XY, CD, and SV-A designed the experiments, analyzed the data, and wrote the manuscript. XY contributed to most of the experiments and figures. MV and L-OE contributed to mass photometry analysis and the figures. CD contributed to *in vitro* phosphorylation assays using radioactive-labeled ATP. WB and US contributed to protein MS analysis and the figures. All authors contributed to the article and approved the submitted version.

## Conflict of Interest

The authors declare that the research was conducted in the absence of any commercial or financial relationships that could be construed as a potential conflict of interest.

## Footnotes

1 http://www.maxquant.org
