# Corrigendum: The Phosphatase PP2A Interacts With ArnA and ArnB to Regulate the Oligomeric State and the Stability of the ArnA/B Complex

**DOI:** 10.3389/fmicb.2020.608420

**Published:** 2020-10-30

**Authors:** Xing Ye, Marian Samuel Vogt, Chris van der Does, Wolfgang Bildl, Uwe Schulte, Lars-Oliver Essen, Sonja-Verena Albers

**Affiliations:** ^1^Molecular Biology of Archaea, Institute of Biology II, University of Freiburg, Freiburg, Germany; ^2^Department of Chemistry, Philipps University Marburg, Marburg, Germany; ^3^Institute of Physiology, Faculty of Medicine, University of Freiburg, Freiburg, Germany; ^4^Center for Biological Signaling Studies (BIOSS), Freiburg, Germany; ^5^Center for Integrative Signaling Studies (CIBSS), Freiburg, Germany; ^6^Loewe Center for Synthetic Microbiology, Marburg, Germany

**Keywords:** Crenarchaea, archaellum, archaellum regulation, protein phosphorylation, protein phosphatases, protein interaction

In the original article, there was a mistake in the motility assay in Figure 1B for the PP2AHA mutant. The corrected Figure 1 appears below.

**Figure 1:**
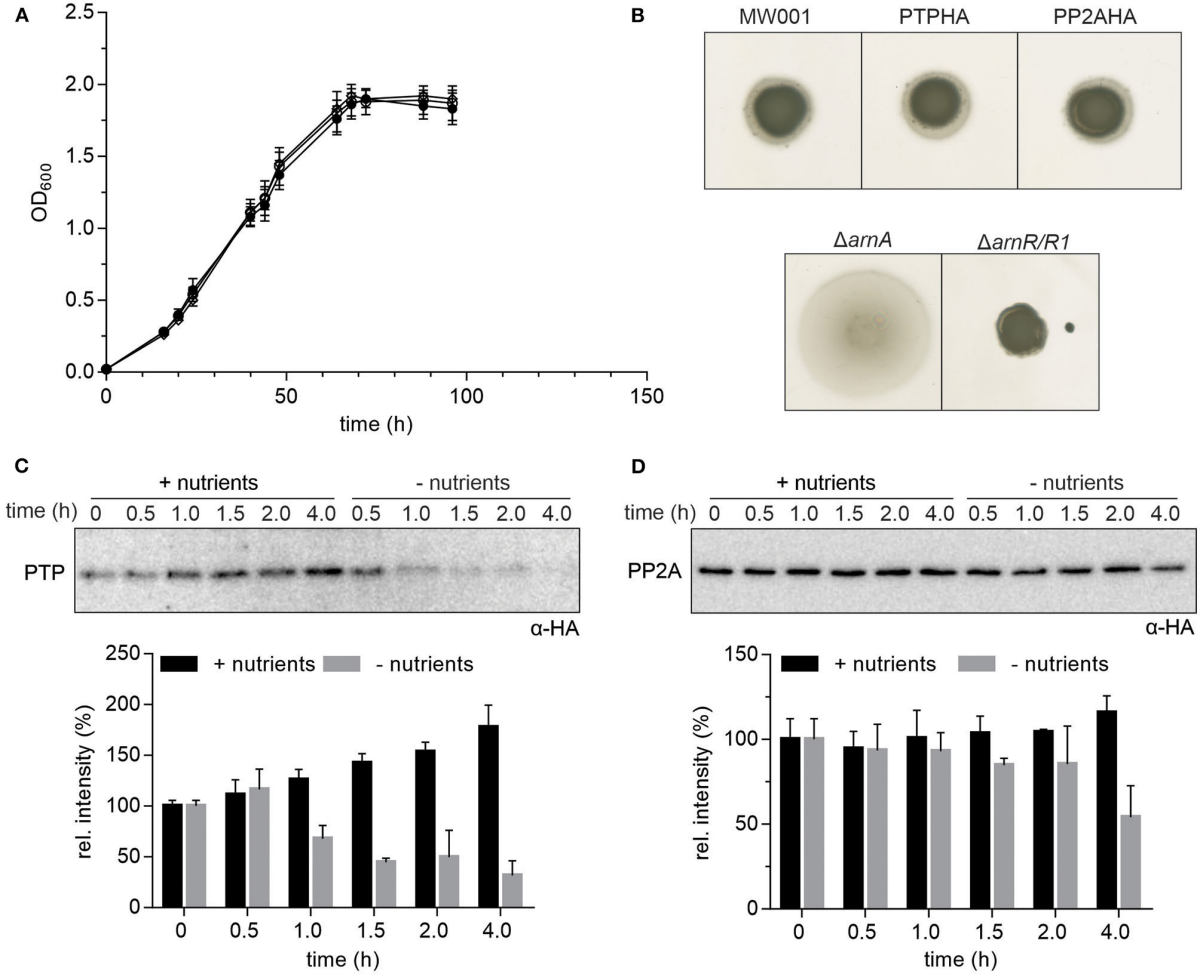
Expression of HA-tagged PP2A and PTP. **(A)** Growth curves of *S. acidocaldarius* cells grown in nutrient rich medium at 75°C were obtained for the parental MW001 strain (black solid circle) and the stains containing HA-tagged PP2A (black open square) and PTP (black open circle). The OD_600_ of each strain was measured at the indicated time points. The average of three independent experiments is shown. **(B)** For motility assays, exponentially growing *S. acidocaldarius* cultures of around OD_600_ 0.4 were spotted on semi-solid gelrite plates with 0.005% (w/v) NZ-amine and incubated at 75°C for 5 days before scanning the plates. The motility assays were repeated with three biological and six technical replicates. A representative experiment is shown. **(C)** Expression of HA-tagged PTP and **(D)** PP2A in *S. acidocaldarius*. PTPHA and PP2AHA mutants were grown in nutrient rich and starvation medium for 4 h. Samples were collected at different time points (0, 0.5, 1.0, 1.5, 2.0, and 4.0 h) and analyzed by Western blotting with α-HA. Representative Western blots are shown. A quantification of three independent experiments is shown below the blots.

The authors apologize for this error and state that this does not change the scientific conclusions of the article in any way. The original article has been updated.

