# Supplementary material for: The Phosphatase PP2A Interacts With ArnA and ArnB to Regulate the Oligomeric State and the Stability of the ArnA/B Complex
Source: Front Microbiol. 2020 Aug 21;11:1849. doi: 10.3389/fmicb.2020.01849 (PMC7472852; doi:10.3389/fmicb.2020.01849)
Supplement: Supplementary file 2 [file Data_Sheet_1.PDF]

Supplemental Material to

The phosphatase PP2A interacts with ArnA and ArnB to regulate the oligomeric state and the stability of the ArnA/B complex

Xing Ye[1], Marian Samuel Vogt[2], Chris van der Does[1], Wolfgang Bildl[3], Uwe Schulte[3,4,5], Lars-Oliver Essen[2,6] and Sonja-Verena Albers[1,4*]

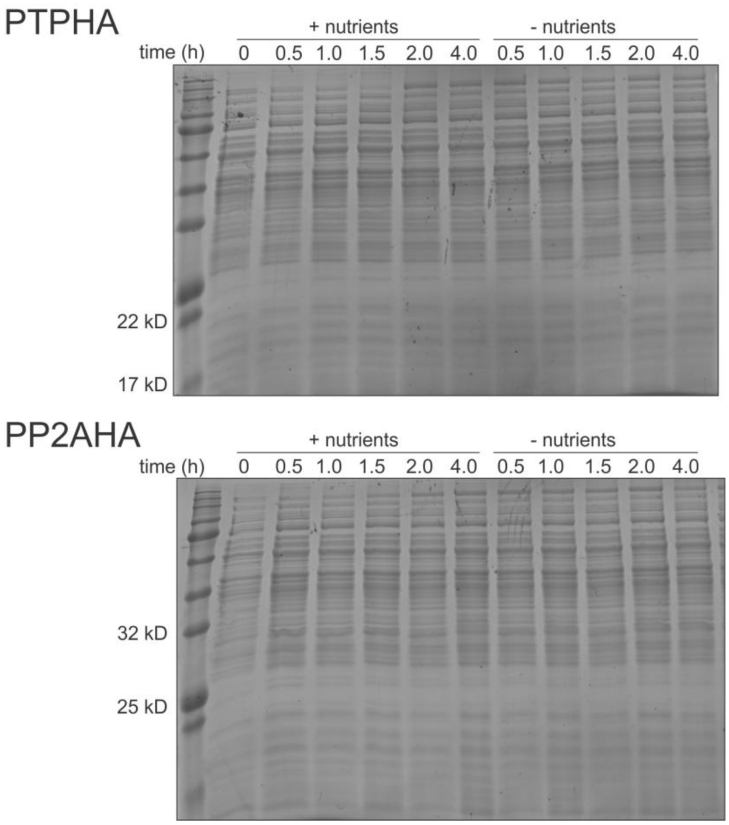

Figure S1 Protein loading controls. PTPHA and PP2AHA mutants were grown in nutrient rich and starvation medium for 4 h. Samples were collected at different time points (0 h, 0.5 h, 1.0 h, 1.5 h, 2.0 h and 4.0 h) and loaded by SDS-PAGE. The coomassie-stained gels which were used as a control for equal loading of the experiments depicted in Figure 1C and 1D are shown.

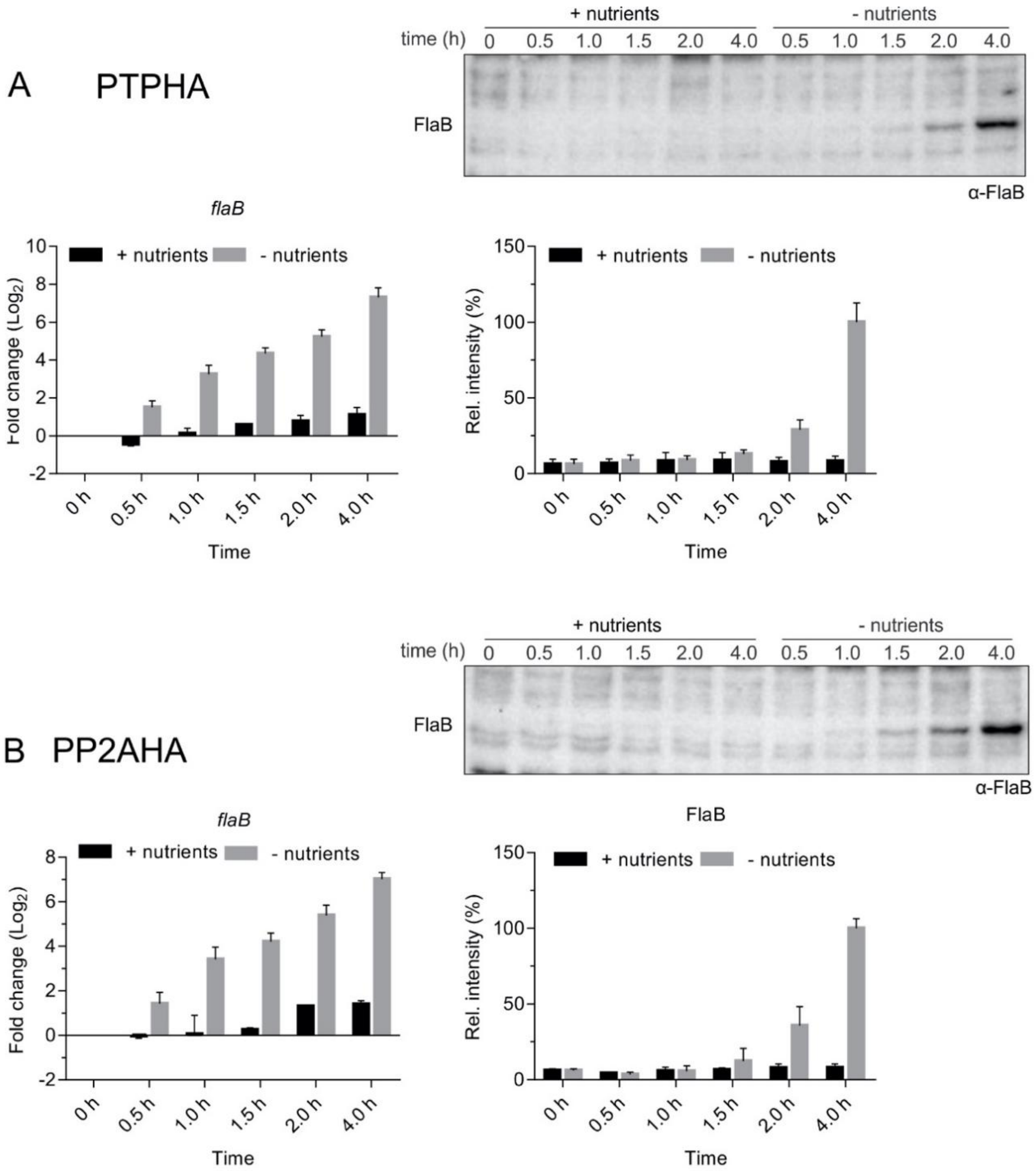

Figure S2 Expression of *flaB* on RNA and protein level in PTPHA and PP2AHA mutants. PTPHA and PP2AHA mutants were grown in nutrient rich and starvation medium for 4 h. Samples were collected at different time points (0 h, 0.5 h, 1.0 h, 1.5h, 2.0 h and 4.0 h) and analyzed by qRT-PCR and Western blotting analysis. Left panel in (A) and (B), qRT-PCR analysis of *flaB*; Right panel in (A) and (B), Western blotting analysis of FlaB. Relative transcription level was normalized to *secY*. The values represent fold changes (mean ± SD) compared with the control from biological triplicates. Western blotting analysis was quantified and given as means ±SD from biological triplicates.

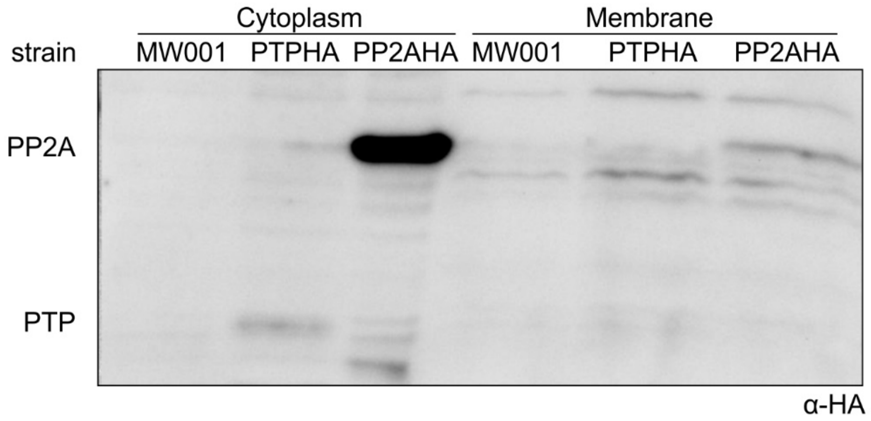

Figure S3 Localization of PTP and PP2A in *S. acidocaldarius* cells. Samples were collected after 0.5 h growth in nutrient starvation medium. Ultracentrifugation was performed to separate cytoplasm and membrane fractions that were further analyzed by Western blotting analysis with α-HA antibody.

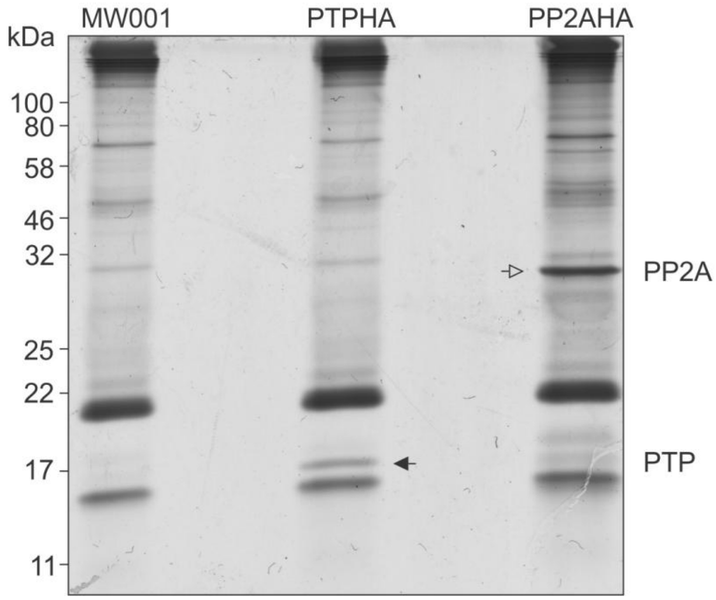

Figure S4 Elution fractions of MW001, PTPHA and PP2AHA from affinity purification with anti-HA magnetic beads were separated on SDS-PAGE. Protein bands were visualized by silver staining. The small filled arrow indicates the position of PTP-HA, and the small non-filled arrow indicates the position of PP2A-HA. Additional bands could be identified at the theoretical weights of a conserved putative ATP/GTP binding protein (Saci_1281, 28.4 kDa), a universal stress protein (Saci_0887, 14.1 kDa) and the archaellum regulators ArnA (22.9 kDa) and ArnB (42.8kDa).

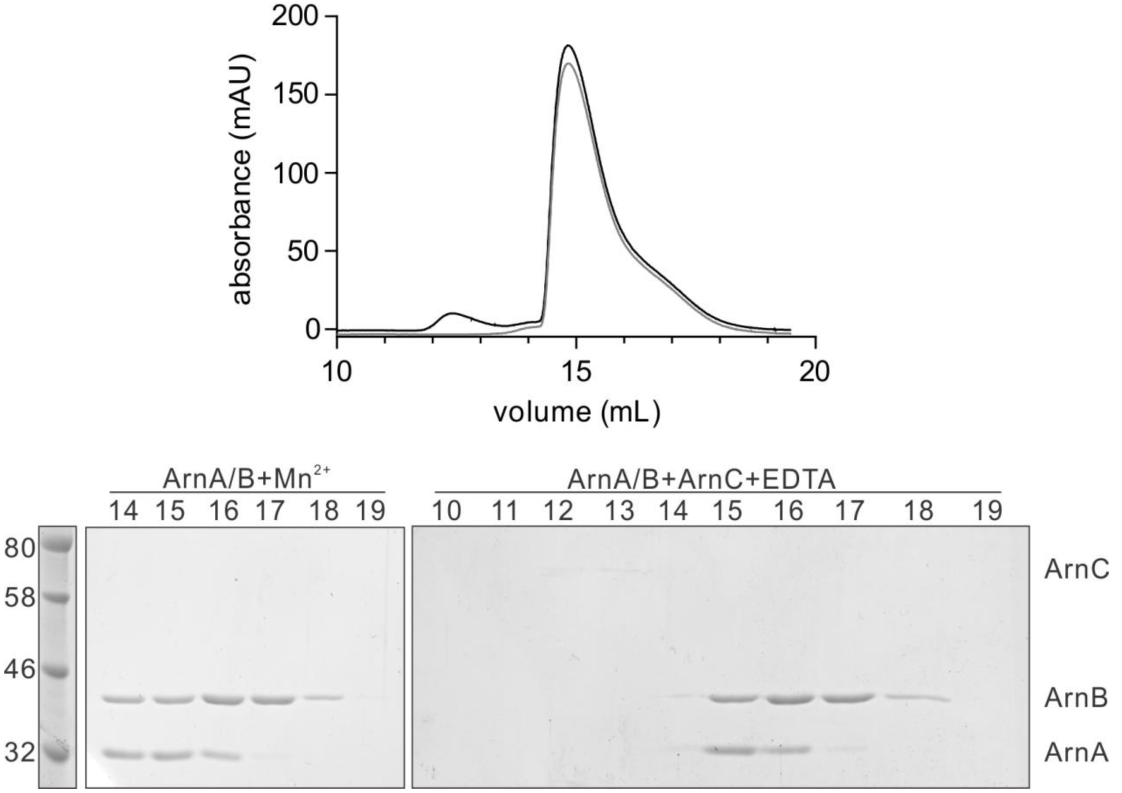

Figure S5 ArnA/B complex was not oligomerized in the presence of ArnC and ATP and absence of Mn^{2+}. The formed ArnA/B complex was incubated with ATP and Mn^{2+} (grey line) or in the presence of ArnC and EDTA (black line) at 55 °C for 30 min and was loaded on a Superdex 200 increase (10/300) size exclusion column. The upper panel depicts the elution pattern observed at 280 nm. Elution fractions were separated on SDS-PAGE and analyzed by Coomassie staining. The SDS-PAGE of the protein fractions around the peaks observed are shown in the lower panel.

Table S1 Strains and plasmids in this study

Strains/plasmids	Genotype	Source/Reference
**Strains**		
Escherichia coli		
Top10	F- *mcrA Δ(mrr-hsdRMS-mcrBC) φ80lacZΔM15 ΔlacX74 nupG recA1 araD139 Δ(ara-leu)7697 galE15 galK16 rpsL(Str^R) endA1 λ$^-$*	Invitrogen
ER1821	λ- *F- glnX44 e14- (McrA-) rfbD1 endA1 thi-1 Δ(yjiT-opgB)114::IS10 (EcoKI R- M- McrBC- Mrr-) + rpoS393(am) creC510 lrhA::IS3 ydeN::IS10*	New England Biolabs
Rosetta (DE3) pLysS	F$^-$ *ompT hsdS*$_B$(r$_B^-$ m$_B^-$) *gal dcm* (DE3) containing the pLysSRARE plasmid (Cam^R)	Novagen
Sulfolobus acidocaldarius		
MW001	*Sulfolobus acidocaldarius* DSM639 Δ*pyrE*	(Wagner et al., 2012)
MW351	MW001 Δ*saci1210* (Δ*arnA*)	(Reimann et al., 2012)
MW332	MW001 Δ*saci_1171Δsaci_1180 (ΔarnR ΔarnR1)*	(Lassak et al., 2013)
MW801	Chromosomally HA-tagged *saci0884* (*saci_pp2a*) gene at the C-terminus	This study
MW802	Chromosomally HA-tagged *saci0545* (*saci_ptp*) gene at the C-terminus	This study
**Plasmids**		
pSVA407	Gene targeting plasmid, pGEM-T Easy backbone, *pyrEFSSO* and *lacSSSO* cassette; single crossover method	(Wagner et al., 2012)
pSVA5102	*saci0884* (*saci_pp2a*) with HA tag in C-terminal, cloned into pSVA407 using *Nco*I and *Bam*HI	This study
pSVA5103	*saci0545* (*saci_ptp*) with HA tag in C-terminal, cloned into pSVA407 using *Nco*I and *Bam*HI	This study
pSVA1009	*saci1193* (*arnC*) with N-terminal His-tag cloned into pETDuet-1 with *Bcl*I/*Bam*HI and *Pst*I	(Reimann et al., 2012)
pSVA1036	*arnB* with C-termial His-tag cloned into pETDuet-1 with *Nco*I and *Bam*HI	(Reimann et al., 2012)
pSVA1037	*saci_pp2a* with C-termial His-tag cloned into pETDuet-1 with *Nco*I and *Bam*HI	(Reimann et al., 2012)
p7XC3H	FX cloning expression plasmid with C-term His tag	(Geertsma and Dutzler, 2011)
p7XC3S	FX cloning expression plasmid with C-term Strep tag	(Quax et al., 2018)
p7XNS3	FX cloning expression plasmid N-term Strep tag	This study
pSVA5131	*arnA* with N-terminal HA tag and C-terminal His tag cloned into p7XC3H by FX cloning method	This study

pSVA5136	*arnA* with N-terminal Strep tag cloned into p7XNS3 by FX cloning method	This study
pSVA5137	*arnB* with C-terminal Strep tag cloned into p7XCS3 by FX cloning method	This study

Table S2 Primers used in this study

Primer name	Sequence (5´- 3´)	Purpose
	**primers for pSVA5102**	
7300	GAGCCATGGGTGAACATTGAAGAAACGTAT	*saci0884-HA* upstr fw
7301	TTACGCGTAGTCCGGAACGTCATACGGGTACT CGAGCGAACCTACTATCTCTTCTATTAGTTG	*saci0884-HA* upstr rev ol
7302	GGTTCGCTCGAGTACCCGTATGACGTTCCGGA CTACGCGTAACAGACAAAAAATAAAAAGACG	*saci0884-HA* downstr fw ol
7303	GAGGGATCCAGGTGTTCTCGCTGACCCATC	*saci0884-HA* downstr rev
1601	TTCCTGCCCACTGATATTCC	*saci0884-HA* check primer fw
1602	CGGTTGGTTAAATCAATTAG	*saci0884-HA* check primer rev
	**primers for pSVA5103**	
7304	GAGCCATGGGAACAGCGGATCTTCAGAGT	*saci0545-HA* upstr fw
7305	CGTAGTCCGGAACGTCATACGGGTATAGTATC TTCCATTTATCTTTCATCTTTTC	*saci0545-HA* upstr rev ol
7306	GTATGACGTTCCGGACTACGCGTAAATGGAA GATTTTATGATAGAATTTCTTTC	*saci0545-HA* downstr fw ol
7307	GAGGGATCCAGCGACTCCGATAGTAGGTTTG	*saci0545-HA* downstr rev
1553	AACTCATAGCGTGAGATCC	*saci0545-HA* check primer fw
1554	ATCCAGCTAATGCATGTTCC	*saci0545-HA* check primer rev
1391	GTAGTCCGGAACGTCATAC	HA tag primer rev
	**primers for pSVA5131**	
9137	ATATATGCTCTTCTAGTTACCCATATGACGTT CCGGACTACGCG	*saci1210 expr fw*
	**primers for pSVA5136**	
9121	ATATATGCTCTTCTAGTACGTGGAAATGTAAT TTATGCGGTTAT	*saci1210* expr fw
9122	TATATAGCTCTTCATGCCTCCTTTAATATTCGT ACTATTGTCTG	*saci1210* expr rev
	**primers for pSVA5137**	
7366	ATATATGCTCTTCTAGTACCATATCAGTTAAA GCCGAATTAAGT	*saci1211* expr fw
7367	TATATAGCTCTTCATGCAGACCTCAACTTCTT AGTAACTTCACT	*saci1211* expr rev
	**primers qRT-PCR**	
7314	TTGTCCCGGTTCTTCCTATG	*ptp*-qRT-PCR-fw
7315	GCCGGAAGAGTTTGAGATTG	*ptp*-qRT-PCR-rev
5411	GAATCATGAAAGTCCACTTACAAAC	*pp2a*-qRT-PCR-fw
5412	AAACCTCCATGCATACAAAG	*pp2a* -qRT-PCR-rev
1480	CCTGCAACATCTATCCATAACATACCGA	*secY*-qRT-PCR-fw
1481	CCTCATAGTGTATATGCTTTAGTAGTAG	*secY* -qRT-PCR-rev

1424	ACTGCGTCTACTGCGTTATCTTTATC	*flaB*-qRT-PCR-fw
1425	GGAGATAAGTCTACACTAGATACACCAGAA	*flaB*-qRT-PCR-rev

References Supplementary Information

Geertsma, E. R., and Dutzler, R. (2011). A versatile and efficient high-throughput cloning tool for structural biology. *Biochemistry* 50, 3272–3278.

Lassak, K., Peeters, E., Wróbel, S., and Albers, S. V. (2013). The one-component system ArnR: A membrane-bound activator of the crenarchaeal archaellum. *Mol. Microbiol.* 88. doi:10.1111/mmi.12173.

Quax, T. E. F., Altegoer, F., Rossi, F., Li, Z., Rodriguez-Franco, M., Kraus, F., et al. (2018). Structure and function of the archaeal response regulator CheY. *Proc. Natl. Acad. Sci.* 115, E1259–E1268.

Reimann, J., Lassak, K., Khadouma, S., Ettema, T. J. G., Yang, N., Driessen, A. J. M., et al. (2012). Regulation of archaella expression by the FHA and von Willebrand domain-containing proteins ArnA and ArnB in Sulfolobus acidocaldarius. 86, 24–36. doi:10.1111/j.1365-2958.2012.08186.x.

Wagner, M., Wolferen, M. Van, Wagner, A., Lassak, K., Meyer, B. H., Reimann, J., et al. (2012). Versatile genetic tool box for the crenarchaeote Sulfolobus acidocaldarius. 3, 1–12. doi:10.3389/fmicb.2012.00214.
